# Silymarin PlantCrystals for Improved Dermal Drug Delivery

**DOI:** 10.3390/bioengineering12121331

**Published:** 2025-12-05

**Authors:** Tehseen Sehra, Muzn Alkhaldi, Cornelia M. Keck

**Affiliations:** 1 Department of Pharmaceutics and Biopharmaceutics, Philipps-Universität Marburg, Robert-Koch-Str. 4, 35037 Marburg, Germany; sehartehseen@gmail.com (T.S.);; 2 Institute of Pharmacy, Faculty of Pharmaceutical and Allied Health Sciences, Lahore College for Women University, Lahore 54000, Pakistan

**Keywords:** skin, dermal drug delivery, nanocarriers, PlantCrystals, extracellular vesicles, ex vivo skin model, poorly soluble compounds

## Abstract

**Background/Objectives:** PlantCrystals (PCs) are submicron particles derived from plants or parts of plants that can be produced by bead milling and/or high-pressure homogenization. Previous studies suggested improved dermal drug delivery of lipophilic active ingredients (API), which was explained by the formation of extracellular vesicles (EVs) during the production of PCs. The aim of this study was to investigate the suitability of PCs for enhancing the dermal penetration efficacy of different types of APIs. **Methods:** For this purpose, hydrophilic, lipophilic, and poorly water-soluble API-surrogates were loaded into PCs, and the dermal penetration efficacy, as well as the skin hydrating properties, were determined with an ex vivo porcine ear model. The penetration efficacy of the API surrogates from the PCs was compared to other formulation principles, e.g., simple API solutions, API loaded into classical EVs, and API added to the PCs after preparation. Silymarin-PCs—unloaded and loaded with API—were obtained by milling milk thistle seeds using small-scale bead milling. The PCs were characterized by size, size distribution, and zeta potential. **Results:** Milling of milk thistle seeds resulted in the formation of submicron particles with sizes of about 300 nm. Loaded PCs had a slightly larger size. Loading API into PCs resulted in improved dermal penetration when compared to the other formulation principles. The effect was most pronounced for the lipophilic API-surrogate (+90%, *p* < 0.001) and least pronounced for the hydrophilic API-surrogate (+2%, *p* > 0.05). The improved penetration of API from PCs can be explained by the formation of EVs during the production of the PCs in which the API is encapsulated. The encapsulation seemed to be highly efficient for the lipophilic API-surrogate, moderate for the poorly soluble API-surrogate, and very limited for the hydrophilic API-surrogate. All formulations increased the skin hydration significantly by about 30–40%. **Conclusions:** Milk thistle seeds are suitable for the production of PCs. These PCs improve skin hydration and enhance the dermal penetration of poorly water soluble and lipophilic APIs. However, they have limited effects on the dermal penetration efficacy of hydrophilic APIs.

## 1. Introduction

**Figure 1:**
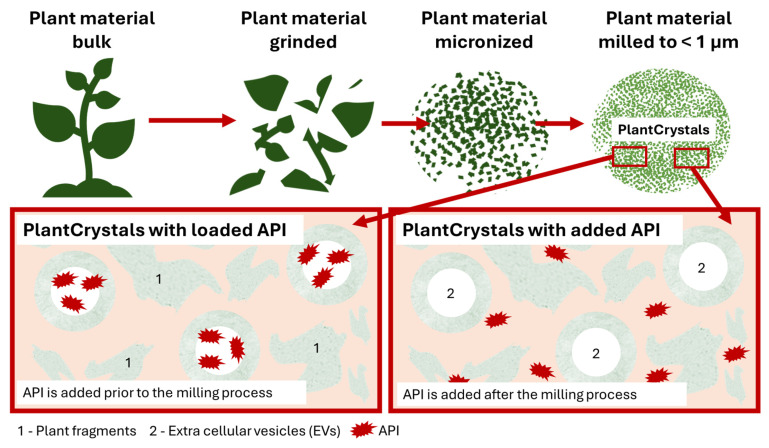
Scheme visualizing the PlantCrystal principle. PlantCrystals are produced by milling plant material to below 1 µm (upper). The milling destroys the plant cells and allows a thorough extraction of the plant components. In addition, extracellular vesicles (EVs) are formed. If APIs are added to the formulations prior to the milling, the API can be encapsulated into the EVs that are formed during the milling (lower left). If the API is added after the milling, the API cannot be encapsulated and remains outside of the EVs (lower-right).

PlantCrystals (PCs) are suspensions of plants or parts of plants that are milled to sizes to below 1 µm. Plant cells typically possess a size of about 10 µm, hence the milling results in the destruction of the plant cells and cell compartments, which allow for a fast and thorough extraction of the plant components without the need of organic solvents. The method creates extracts that are more effective than classical extracts [1,2,3,4]. Furthermore, a recent study also showed that the milling and/or homogenization of plants or plant materials not only destroys the plant cells but also allows for the formation of extracellular vesicles (EVs) [5]. The first study of this kind was performed by producing cucumber-derived PlantCrystals in which a lipophilic active pharmaceutical ingredient (API) surrogate was incorporated. In this study, the formation of EVs during the PlantCrystal production was demonstrated by various techniques, including particle tracking analysis, electron scanning microscopy, atomic force microscopy, and protein quantification and profiling. The bio-efficacy of the API was determined by analyzing the dermal penetration efficacy from the PlantCrystals with the ex vivo porcine ear model. Classical EVs, the API-surrogate in solution, and PlantCrystals to which the API-surrogate was added after the milling process served as benchmark controls. Results demonstrated that the PlantCrystals with EVs only enhanced the dermal penetration of the lipophilic API when it was added to the plant suspension prior to the milling process, whereas the dermal penetration was not enhanced when the API surrogate was added to the plant suspension after the milling. From this it was concluded that the incorporation of the API into EVs only takes place when the API is added to the formulation prior to the milling (Figure 1), which allows the encapsulation of the API into the EVs once they are formed [5].

The second study used soybeans as the source for PlantCrystals and classical EVs and encapsulated a poorly soluble BCS class IV API into the PlantCrystals and EVs. Results demonstrated—as already seen for the lipophilic API surrogate in the first study—that PlantCrystals can increase the dermal penetration efficacy of the API surrogate; however, this is possible only if the API is added to the plant suspension prior to the milling process. Adding the API to the suspension after the milling did not lead to an improved performance.

**Figure 2:**
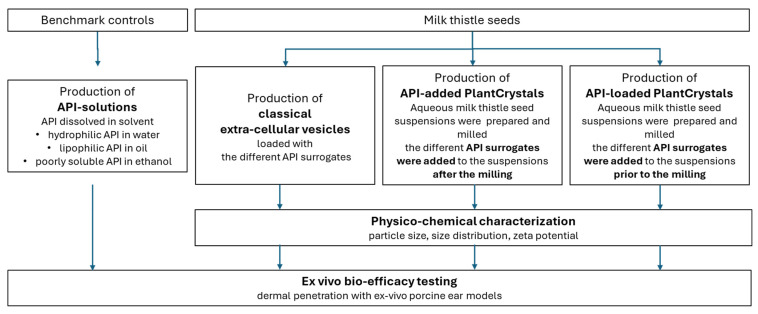
Experimental setup of the study.

The results of these two studies also demonstrated that the PlantCrystal technology can be used for different types of plant material and different types of API (lipophilic and poorly soluble). However, a study that investigates the suitability of the PlantCrystals to enhance the penetration of hydrophilic APIs was not yet conducted, and a study that systematically compares the efficacy of the PlantCrystal technology to enhance the dermal penetration of hydrophilic, lipophilic, and poorly soluble APIs is also not available yet. Therefore, the aim of this study was to answer these scientific questions. In order to fulfill this aim, PlantCrystals were produced from milk thistle seeds. The PlantCrystals were loaded with either a hydrophilic, lipophilic, or poorly water-soluble API surrogate, and their physico-chemical properties (size, zeta potential) were determined. The dermal penetration efficacy of the different API surrogates was also determined and compared to other formulations (API solutions, classical EVs, PlantCrystals to which the APIs were added after production; Figure 2).

## 2. Materials and Methods

### 2.1. Materials

Milk thistle seeds (*Silybum marianum* (L.) seeds) were selected as plant material and source for the PlantCrystals. Milk thistle seeds are well known for their health beneficial properties [6]. The seeds contain silymarin and other antioxidants, as well as omega-6 fatty acids, which are not only beneficial for stimulating liver regeneration but also for the maintenance and restoration of the skin barrier [7,8]. Therefore, milk thistle seeds and PlantCrystals made from them were considered to have a “2 in 1” benefit. First, they were expected to act as EV donors that can encapsulate different kinds of active compounds, and second, they hold the potential to simultaneously improve skin functionality, well-being, and health. The milk thistle seeds were obtained in food grade from Alpi Investment Ltd. (Sofia, Bulgaria).

Polysorbate 80 (Kolliphore^®^ 80) was used as surfactant and was acquired from BASF SF (Ludwigshafen, Germany). Phosphate-buffer saline (PBS, pH 7.4 at 25 °C) and purified water PURELAB^®^ Flex 2 (ELGA LabWater, Veolia Water Technologies Deutschland GmbH, Celle, Germany) were freshly prepared prior to each experiment. The API surrogates were sodium fluorescein ((SF) from Carl Roth GmbH (Karlsruhe, Germany) as hydrophilic API surrogate, Nile red ((NR) from Sigma-Aldrich Chemie GmbH (Steinheim, Germany) as lipophilic API-surrogate, and curcumin (Cur) from Receptura Apotheke, Cornelius-Apothekenbetriebs-OHG (Frankfurt am Main, Germany) as poorly water-soluble API surrogate.

### 2.2. Methods

#### 2.2.1. Production of Classical Plant-Derived EVs (PEVs)

Classical plant-derived EVs from milk thistle were produced after previously developed protocols for the production of cucumber and soybean derived PEVs [5,9]. For this, in the first step, the milk thistle seeds (≈40 g) were ground in a high-speed multifunction grinder (Electric Grain Mill 850W 28000RPM, Vayotoy, China) and sieved using a 30-mesh size and then a 100-mesh size. To 30 g of powdered milk thistle seeds, filtered (0.22 µm) PBS (pH 7.4) was added to make a total of 240 g. This suspension was then subjected to further grinding in an electric mixer. To isolate PEVs of milk thistle, differential ultracentrifugation (DUC) was carried out using a Sorvall™ centrifuge (RC 6+ Centrifuge, Fisher Scientific GmbH, Schwerte, Germany). A sequential centrifugal protocol was carried out at relative centrifugal forces (RCF) of 400× *g*, 800× *g*, 2000× *g*, and 15,000× *g*, each for 30 min at 4 °C with an F21S-8 × 50y rotor. Afterwards, the resultant supernatant was collected cautiously and filtered using filter paper of 0.45 µm, whereas the pellets were discarded. The centrifugation of the filtered supernatant was executed in a Sorvall™ ultracentrifuge (MTX 150 Micro-Ultracentrifuge, Fisher Scientific GmbH, Schwerte, Germany) at 120,000× *g* at 4 °C for 60 min with a S80-AT3 rotor. At this stage, the supernatants were carefully discarded leaving pellets undisturbed and the process was repeated by adding more samples to the same tubes until the whole sample was furnished. The pellets from all the tubes, which are anticipated to contain milk thistle (silymarin) PEVs, were collected in one Eppendorf tube. All the tubes were rinsed with 50 µL of PBS and added to the Eppendorf tube obtained at the end. The thus obtained classical silymarin EVs were resuspended with PBS, vortexed for 20 min, and stored at 4 °C until further analysis and use.

The loading of the classical EVs with the different API surrogates was performed in the next step by adding the required amount of either SF, NR, or curcumin to aliquots of the unloaded EVs where the concentration of curcumin was 0.5% *w*/*w* and 0.005% *w*/*w* for both NR and SF in the final formulations. The obtained mixtures were ultrasonicated for 30 min, vortexed gently for 15 min, and then incubated in the dark for 30 min at room temperature. Ultracentrifugation was then carried out at 120,000× *g* for 60 min at 4 °C. The supernatant, holding excess API surrogate, was discarded. The pellets containing API-loaded classical EVs were collected from all tubes, suspended in PBS (≈200 µL), vortexed for 20 min, and stored at 4 °C until further analysis and use.

#### 2.2.2. Production of PlantCrystals (PCs)

In the first step, milk thistle seeds were ground in a high-speed multifunction grinder (Electric Grain Mill 850W 28000RPM, Vayotoy, China) and sieved using a 30-mesh size and then a 100-mesh size. The powder obtained was then subjected to small-scale bead milling, as described previously [5,9,10]. For this, the milk thistle powder (1% *w/w*) was suspended in an aqueous Polysorbate 80 solution (1% *w*/*w*) in a 25 mL Erlenmeyer flask. Yttria-stabilized zirconium oxide beads with a diameter of 1.0–1.2 mm (SiLibeads^®^, Sigmund Lindner GmbH, Warmensteinach, Switzerland) were added to this in a ratio of 60:40 (*v*/*v*) (suspension/bead) along with a magnetic stirring bar (ASTEROID^®^ 25, 2mag AG, Munich, Germany). The assembly was placed on a magnetic stirrer (IKA^®^ RET basic, IKA-Werke GmbH, Staufen, Germany) and milled at 1200 rpm for 8 h in an ice bath.

For the preparation of API-loaded PCs, the required amounts of the API surrogates, i.e., SF (0.005% *w*/*w*), NR (0.005% *w*/*w*), or curcumin (0.5% *w*/*w*), were added to the assemblies prior to the milling. For the production of the API-added PCs, the API surrogates were added to the blank PCs after the milling process (c.f. Figure 2). The obtained formulations were stored at 4 °C until further analysis and use.

#### 2.2.3. Determination of Particle Size and Size Distribution

Analysis of particle size and particle size distribution was carried out using photon correlation spectroscopy (PCS), laser diffractometry (LD), light microscopy (LM), and epifluorescence microscopy (FM). PSC (also known as dynamic light scattering—DLS) was used to determine the hydrodynamic diameter (z-average) and polydispersity index (PDI) using a Zetasizer Nano ZS (Malvern Panalytical GmbH, Kassel, Germany). The formulations were first diluted with purified water and the measurements were carried out at 20 °C. As PlantCrystals are not monodisperse but polydisperse formulations, the data were analyzed using the general-purpose mode in the built-in software. LD was used as an additional technique for size analysis to identify possible larger particles present in the formulations. This is important as larger particles cannot be detected by PCS but can have a significant impact on the stability, properties, and overall performance of nanocarrier formulations [11,12,13]. LD analysis was performed with a Mastersizer 3000 (Malvern Panalytical Ltd., Malvern, UK). Data analysis was performed with Mie theory with the real refractive index set to 1.47 and the imaginary refractive index to 0.1. The results are presented in the form of median volume-based diameters (d(v)0.1–d(v)0.99). LM visualizes particles ≥500 nm and it is a simple and swift method to observe possible larger particles within nanocarrier formulations; therefore, it was used to confirm the PCS and LD results. For this, an Olympus BX53 (Olympus Corporation, Tokyo, Japan) fitted with an SC50-CMOS color camera (Olympus Soft Imaging Solutions GmbH, Münster, Germany) was used. FM was used to visualize the distribution of the API surrogates within the formulations. For this, a CKX53 equipped with an Olympus DP22 color camera (Olympus Life Science Solutions GmbH, Hamburg, Germany) was used.

#### 2.2.4. Zeta Potential Analysis

The zeta potential (ZP) of the PEVs and PC formulations was determined with a Zetasizer Nano ZS (Malvern Panalytical, Kassel, Germany). For this, the electrophoretic mobility was first determined via laser Doppler anemometry (LDA), which was then converted to the ZP using the Helmholtz–Smoluchowski equation. For the measurements, the samples were dispersed in purified water with a conductivity adjusted to 50 µS/cm using NaCl. Measurements were performed at 20 °C. The final ZP values are shown as mean [mV] ± standard deviation (SD).

#### 2.2.5. Dermal Penetration Efficacy—Static Model

The dermal penetration was assessed with the ex vivo porcine ear model [14,15]. Fresh pig ears were obtained from a local slaughterhouse, washed, and dabbed dry with a soft tissue. The dorsal side of the porcine ears was used for the penetration experiments. Prior to the experiments, the skin integrity (measured as transepidermal water loss, TEWL) was checked with a Tewameter^®^ TM 300 (Courage-Khazaka electronic GmbH, Köln, Germany), and only ears with TEWL values ≤ 10 g/m^2^/h were used for the studies. In the next step, areas of 2 × 2 cm were marked on intact skin areas without scratches and 30 µL of each formulation, corresponding to an applied dose of 7.5 µL/cm^2^ (finite-dose setup), were applied to the areas with a pipette. The formulations were evenly spread with the pipette tip without applying any pressure or massage. The formulations were then allowed to penetrate into the skin for 2 h at 32 °C. After this, the formulations were gently washed off, and the ears were dabbed dry. Punch biopsies (diameter 15 mm) of the treated skin areas were taken, embedded in Tissue-Tek^®^ (Sakura Finetek Europe BV, Alphen aan den Rijn, The Netherlands), and immediately frozen at −40 °C. Untreated skin sections were also taken and served as controls. Each formulation was tested in triplicate on 3 different, independent ears (ears from different donors). For further analysis, the punch biopsies were cut into 20 µm thick vertical skin sections (cryomicrotome, Model 2700, Reichert-Jung, Nußloch, Germany) and placed on objective slides. Inverted epifluorescence microscopy (Olympus CKX53 with an Olympus DP22 color camera; Olympus Life Science Solutions GmbH, Hamburg, Germany) was used to obtain representative images of each formulation. The microscope settings were kept constant throughout the entire study and were set to 50% intensity of the fluorescence light source and 50 ms exposure time.

The FITC (for SF and Cur) and TRITC (for NR) filter blocks were used for imaging with the following filter setups: Cur and SF: excitation 460–500 nm, dichroic mirror 500 nm, emission > 500 nm (LP); nile red: excitation 540–560 nm (BP), dichroic mirror 570 nm, emission > 580 nm (LP). From each biopsy, 40 pictures were taken, resulting in 120 images (*n* = 3) for each formulation tested. In the next step, the images were analyzed using ImageJ (version 1.8.0). The amount of penetrated API was analyzed semi-quantitatively following a previously described protocol [14,15]. Briefly, an automated thresholding algorithm was applied to each image to remove skin autofluorescence. The remaining pixels in the images served as a semi-quantitative measure of the amount of penetrated API (penetration efficacy given as mean gray value per pixel, MGV/px). The mean penetration depth (MPD) of the API into the skin was determined by measuring the distance between the skin surface and the most distant pixel, using the software’s scale bar. Similarly, the stratum corneum thickness (SCT) was determined from the original images. The SCT is a sensitive measure of skin hydration and was therefore used to assess the influence of the different formulations on skin hydration [14,15].

#### 2.2.6. Dermal Penetration Efficacy—Dynamic Model

The kinetic penetration profiles of the different formulations were evaluated over 24 h. For this, skin (full thickness, ~1 mm) was removed from the dorsal side above the upper cartilage of porcine ears (*n* = 3) with a surgical scalpel blade. Scratch- and bruise-free skin areas were punched (Ø 18 mm), carefully spread, and placed at the bottom of a 24-well plate. Five microliters of each formulation were applied to the different skin areas and controls without skin but with formulations only were also placed in separate wells. The plates were covered and then placed in a microplate reader (Infinite^®^ 200 PRO, Tecan Group Ltd., Männedorf, Switzerland) for analysis over 24 h. The temperature was maintained at 32 °C, and bottom readings were performed every 15 min for each sample. Depending on the API surrogates, the bottom readings were set at different wavelengths (Table 1).

The quantification of the amount penetrated over 24 h was performed with the help of regression equations, generated with the help of calibration curves. The experiments were carried out in triplicate.

The classical formulations were solutions of the API surrogates. Specifically, 0.005% (*w*/*w*) SF was dissolved in water, 0.005% (*w*/*w*) NR was dissolved in medium-chain triglycerides (MCT), and 0.5% (*w*/*w*) curcumin was dissolved in ethanol (96% *v*/*v*). These solutions were used as benchmark controls and were applied to the skin in the same way as all other formulations in this study.

#### 2.2.7. Statistical Analysis

JASP software (version 0.18.3.0, University of Amsterdam, Amsterdam, The Netherlands) was used to generate descriptive statistics and compare the mean values, whereby the test for normality was conducted with the Shapiro–Wilk test and homogeneity of variance with Levene’s test. The comparison of the mean values was performed with one-way ANOVA when the data were normally distributed. Welch’s correction was taken into account in cases where the variance exhibited heterogeneity. Kruskal–Wallis analysis of variance was performed for non-parametric data sets and appropriate post hoc tests (Games–Howell, Dunn’s, and Dunnett) were performed to gain detailed understanding of the differences between the different mean parameters. All *p*-values < 0.05 were considered to be statistically significant. Detailed results of the statistical outcomes are summarized in the Supplementary Material.

## 3. Results

### 3.1. Production and Characterization of PlantCrystals and Classical PEVs

**Figure 3:**
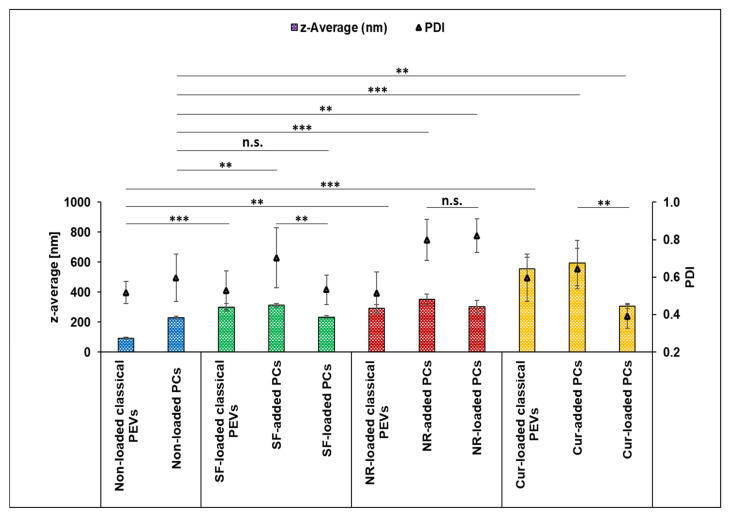
Size analysis (DLS data) of the different formulations. Statistical comparisons were performed on the particle size data (z-average) (** *p* < 0.01, *** *p* < 0.001, n.s.: non-significant).

**Figure 4:**
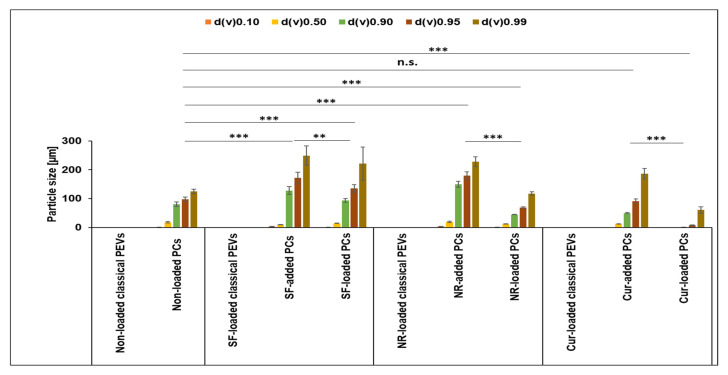
Size analysis (LD data) of different PC formulations. Statistical comparisons were carried out for d(v) 0.95 (** *p* < 0.01, *** *p* < 0.001, n.s.: non-significant).

**Figure 5:**
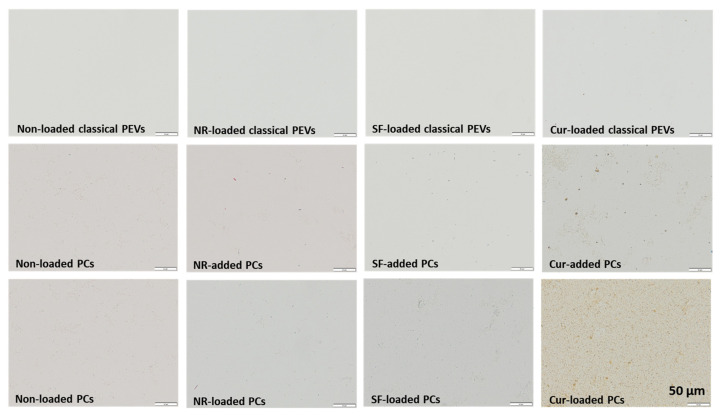
Light microscopic images of the different formulations to visualize possible larger sized micrometer particles within the different formulations. Scale bars represent 50 µm.

**Figure 6:**
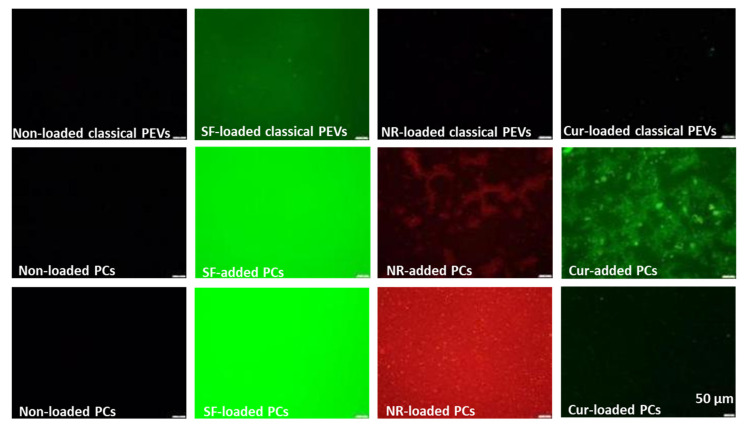
Images taken with an inverted fluorescence microscope to visualize the distribution of the different fluorescent API surrogates within the different formulations. Scale bars represent 50 µm.

The different formulations were successfully produced and characterized regarding their physico-chemical properties (Figure 3, Figure 4, Figure 5 and Figure 6). The average particle size of the non-loaded classical PEVs was about 100 nm, and when these particles were loaded with SF or NR, their hydrodynamic diameters increased significantly to about 300 nm (Figure 3).

When loaded with Cur, the size of classical PEVs increased to about 550 nm (Figure 3). The PdI value of the non-loaded EVs was very high (0.5) and further increased to up to 0.6 when loaded with the different API surrogates. The data indicate that the formulations contained some larger particles and/or agglomerates. LD measurements from the PEVs were not possible, because the scattering intensity—even after adding larger amounts of sample—remained too low to conduct a valid measurement. This can happen if the number of particles within a sample is very low and/or if the refractive index of the particles is close to that of water. Both phenomena can be considered to be relevant for the PEVs. However, light microscopy (Figure 5) and fluorescence microscopy (Figure 6) can also be used to visualize larger particles within a small-sized sample. These methods are simple but reliable. In case of the PEVs, no larger particles were visible in the images (Figure 5 and Figure 6). Hence, all PEVs can be considered to possess nanosized particles well below 1 µm. The non-loaded PCs possessed a size of about 330 nm. The much larger size of the PlantCrystal formulation compared to the classical PEVs was also confirmed by LD measurements, which showed the presence of micrometer-sized particles up to 100 µm (Figure 4).

These large particles were not visible under the light microscope, which only showed a few small micrometer-sized particles (Figure 5). The differences in LD and light microscopy indicate that the large particles seen in LD are loose agglomerates that might have formed during LD measurements.

The larger particles of the unloaded PlantCrystals are due to the presence of other plant parts and fragments that were separated from the classical PEVs during the filtration step. These results are in line with previous studies that also showed a rather broader size distribution of PlantCrystals [16,17,18]. The size of the PCs further increased and the size distribution became even broader upon the addition or loading of SF, NR, and Cur (Figure 3, Figure 4 and Figure 5).

The largest particles and the broadest size distribution were obtained when the API surrogates were added to the PlantCrystals after production. The results indicate that the loading or addition of the API surrogate changes the particles and the interaction with the API surrogate. It also demonstrates that the method of PlantCrystal production is important for the distribution of the API surrogate within the formulation. This estimation is confirmed by the images obtained from the fluorescence microscope, which clearly show a different distribution of the lipophilic and poorly soluble API surrogates (Figure 6).

These differences were expected because it is hypothesized that the API can be incorporated into the EVs when it is added to the formulation prior to production, because the EVs are believed to form during production. If the API is added after the production and, with this, after the formation of the EVs, the API is very unlikely to be incorporated into the EVs and is considered to remain outside the EVs (c.f. Figure 1). Hence, the data obtained here confirm this theory and substantiate the hypothesis that EVs are formed during the production of the PlantCrystals.

Interestingly, there are no differences in API-surrogate distribution of the hydrophilic API surrogate (Figure 6—left). Here, the distribution seems to be homogeneous and similar in all formulations produced. This finding suggests that the hydrophilic API surrogate (or parts of it) is not incorporated into the EVs but is rather located as dissolved molecules in the surrounding water phase of the formulations.

Zeta potential measurements were used to understand these results in more detail (Table 2). The zeta potential is a measure of the surface charge of the particles and thus a sensitive parameter when the surface or membrane composition of a vesicle is changed. The PEVs had a ZP of −23 mV and the PlantCrystals had a slightly higher ZP of about −25 mV (Table 2). The increase in ZP is also in line with previous studies and is due to the additional plant parts and components that were isolated from the PEVs during the filtration process but remained in the PlantCrystals [16,17,18]. Loading the PEVs and the PlantCrystals with the hydrophilic API surrogate had almost no influence on the ZP. However, when the hydrophilic API surrogate was added to the PlantCrystals after production, the ZP decreased by about 5 mV. The differences between loaded and added formulations indicate that at least some extent of the hydrophilic API surrogate was encapsulated in the EVs but was not when the API surrogate was added to the PlantCrystals after production. For the lipophilic API surrogate, the mode of loading had no influence on the ZP. In all cases, the addition or loading of NR resulted in a small increase in ZP by about 3 mV. This means it can be hypothesized that NR was completely encapsulated in the PEVs and PCs during production and diffused passively into the EVs when it was added after production. However, the size data and the fluorescence images reveal significant differences between the differently loaded formulations. Therefore, another reason for the similar ZP values might be that the larger particles with possibly different surface structures—which were seen by LD measurements and microscopic analysis—and/or non-dissolved lipophilic NR particles were not measured by the instrument, which is only able to analyze nanosized particles. Hence, results of the ZP measurements might not reflect the different surface charges of all different types of particles within the samples.

However, the most pronounced influence on the ZP was seen when the poorly soluble API surrogate was loaded or added to the particles. Here, in all cases, the ZP was reduced (Table 2). The reason for this is the high amount of curcumin that was added to the formulations. It seems that this amount was too high to be encapsulated into the PEVs, which resulted in the presence of larger curcumin crystals in all samples—as seen by DLS, LD, and LM measurements (c.f. Figure 4, Figure 5 and Figure 6).

Despite the curcumin crystals that are present in all formulations, there are also large differences in size and ZP results between the different formulations. These differences indicate that the mode of API addition during production strongly influences the location of the curcumin. Based on ZP and size measurements, most of the curcumin seems to be encapsulated in the PlantCrystals when it was added prior to the production of the PlantCrystals (PCs-loaded). Least curcumin seems to be encapsulated in the EVs when it was added to the PlantCrystals after their production (PCs-added). The classical PEVs seem to be in between these two formulations (Figure 3, Figure 4, Figure 5 and Figure 6, Table 2).

### 3.2. Dermal Penetration

**Figure 7:**
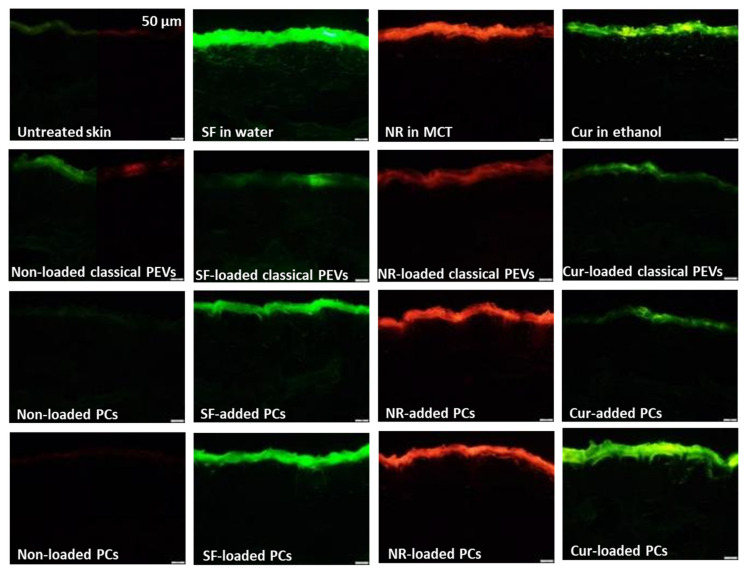
Epifluorescence microscopic images showing vertical skin cuts of the untreated skin and the skin after the application of the different formulations. Scale bars represent 50 µm.

**Figure 8:**
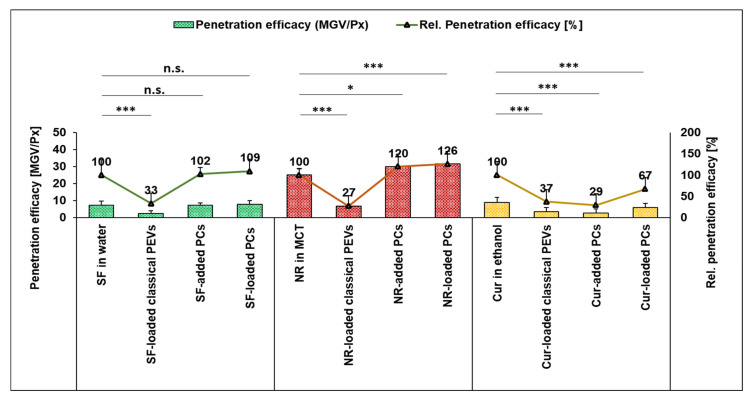
Penetration efficacy (MGV/Px) and rel. penetration efficacy [%] of the different formulations (* *p* < 0.05, *** *p* < 0.001, n.s.: non-significant).

**Figure 9:**
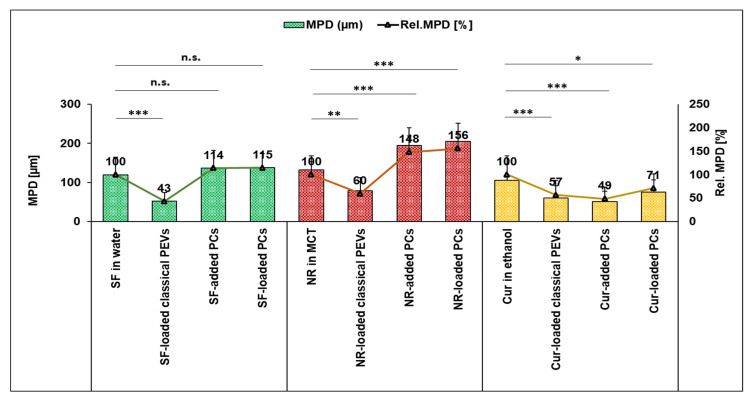
Mean penetration depth (µm) and rel. MPD [%] of the different formulations (* *p* < 0.05, ** *p* < 0.01, *** *p* < 0.001 n.s.: non-significant).

**Figure 10:**
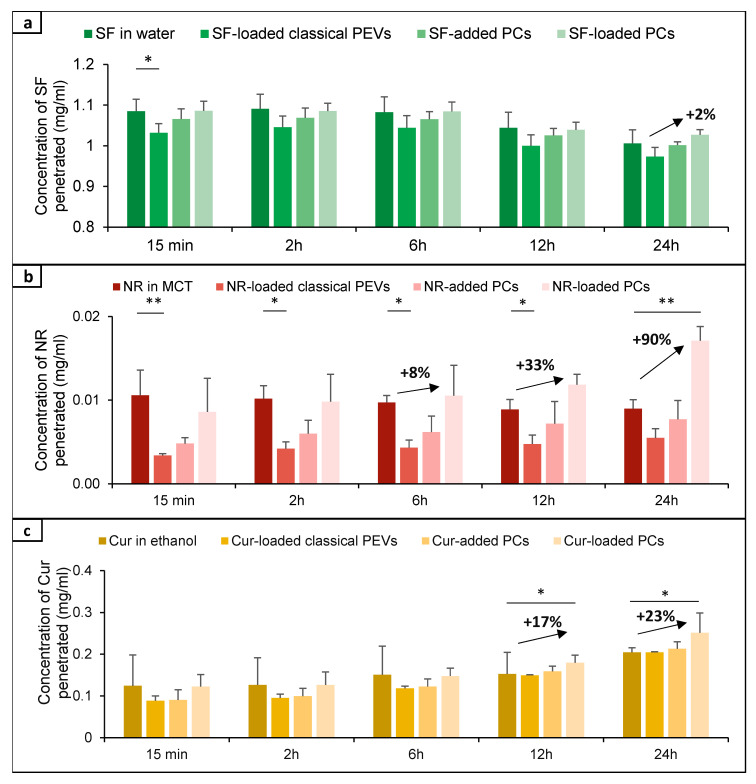
Time-dependent penetration efficacy (mg/ml) and rel. penetration efficacy [%] of the different formulations ((**a**) hydrophilic AI-surrogate, (**b**) lipophilic AI-surrogate, (**c**) poorly soluble AI-surrogate) over 24 h. (* *p* < 0.05, ** *p* < 0.01).

The next step evaluated the bio-efficacy of the different formulations by determining their ex vivo dermal penetration efficacy (Figure 7, Figure 8, Figure 9 and Figure 10). All different types of API surrogates tested confirmed the beneficial effects of the loaded PCs when compared to classical PEVs or to PCs in which the API surrogate was added to the formulation after the production of the PCs (PCs-added).

These results are in line with our previous studies, where a similar trend was shown [5,9]. Hence, this study also supports the previous findings and hypothesis that EVs are created during the milling of plant materials. In addition, this study also confirms that the addition of the API, which is meant to be encapsulated into the EVs, needs to be added prior to the milling process. During milling, the EVs are created and the API can be encapsulated into the EVs efficiently. Addition of the API after milling means the API is added after the EVs have already formed and, therefore, encapsulation of the API is not possible or at least less efficient, which results in less efficient dermal penetration of the API (c.f. Figure 1).

Despite comparing PlantCrystals and classical PEVs, this study also compared the penetration efficacy of the API from PlantCrystals to classical formulations that allow for efficient dermal penetration of the respective API. As classical formulation principle, simple API solutions in which the different API surrogates were dissolved were selected.

Molecules being dissolved in a solvent enter the skin via the solvent-drag mechanism. Hence, after dermal application the solvent (for example water, oil, or organic solvent) penetrates into the skin and drags the molecules dissolved in it with it into the skin [19]. This mechanism is very fast and effective, and thus formulations that allow for such a kind of API uptake were chosen as a simple to prepare, yet effective formulation principle to act as a benchmark control.

The comparison between benchmark controls (API-surrogate solutions), PEVs, and PC formulations shows that the classical PEVs were less efficient than the API solutions (Figure 8 and Figure 9). The reason is probably due to less API being available in the classical PEVs because of the extensive purification process required for their production. Another reason could be a different release profile of the API surrogates, i.e., fast diffusion into the skin from the solutions with the dissolved API molecules and a slower but controlled release from the PEVs and PC formulations. Hence, it was hypothesized that longer penetration times might alter the results and might allow for better penetration of the API surrogates from the PEVs and PC formulations. Therefore, to investigate the influence of time, the dermal penetration of the different formulations was also tested in the kinetic ex vivo model, which allows monitoring the dermal penetration at different time points over 24 h (Figure 10).

The results from the ex vivo kinetic model confirm that a longer penetration time improved the penetration efficacy of the API surrogates from the PEVs and PC formulations compared to the benchmark controls, thus demonstrating a more controlled release of the API surrogates from these formulations. The effects were observed for all types of API surrogates.

The comparison of the data reveals differences between the different formulations and the different types of API surrogates, and it also shows that the data obtained from the static model (Figure 8 and Figure 9) cannot always be correlated with the results seen in the kinetic model (Figure 10). This is reasonable because the static model investigates only one time point (2 h penetration) but yields a detailed information on the penetration of the API surrogate into the skin, which is expressed with two different parameters—the ART value as a semi-quantitative measure of the total amount of penetrated API and the MPD, which indicates how deeply the API penetrated into the skin during this time.

The kinetic model monitors penetration over 24 h and provides measures of how much API penetrated through the skin (full-thickness skin—1 mm) during this time. API that penetrates only into the upper layers of the skin (stratum corneum, epidermis, upper part of the dermis) will not be detected by this method. Therefore, both models provide important but different insights into the dermal penetration of the different APIs from the different formulations.

For the hydrophilic API surrogate, the static model (2 h penetration time) detects least penetration of the API from the PEVs and no statistical differences between the penetration efficacies of the API from the benchmark control (API in water) and the PlantCrystals (Figure 8 and Figure 9). The kinetic model (Figure 10a) shows a less efficient (transdermal) penetration of the API surrogate from the PEVs after a short penetration time. The effect cancels out over time (≥2 h) and results in similarly efficient dermal penetration of the hydrophilic API surrogate from all formulations tested. Hence, both models suggest that PEVs and PC formulations are not beneficial for enhancing the dermal penetration of the hydrophilic API surrogate.

After 2 h penetration time for the lipophilic API surrogate, the static model shows very poor penetration of the API from the PEVs as compared to the benchmark control (API dissolved in MCT oil) and an improved penetration efficacy of the API from the PlantCrystals (Figure 8 and Figure 9). No statistical difference in the penetration efficacy was found between the API-added and API-loaded PlantCrystals. The kinetic model confirmed the limited penetration of the API from the PEVs and showed no differences between benchmark control and PlantCrystals for short and medium penetration times (≤12 h). After longer penetration times (24 h), the amount of transdermally penetrated API was almost two-fold higher from the PlantCrystals (+90%, *p* < 0.01). No differences were found between benchmark control, classical PEVs, and PlantCrystals with added API surrogate (Figure 10b).

The dermal penetration efficacy of the poorly water-soluble API surrogate was less efficient from all PEVs and PC formulations when compared to the benchmark control (curcumin dissolved in ethanol) in the static model after 2 h penetration time (Figure 8 and Figure 9). In the kinetic model, similar trends were seen with shorter penetration times (≤2 h). Long penetration times (≥12 h) showed an improved penetration of the curcumin from the PlantCrystals with loaded API that was further improved after 24 h of penetration (approximately +20%, *p* < 0.01).

With these results, the study demonstrates that the penetration efficacy of the API from PEVs and PCs is complex and not easy to predict, as it is influenced by many interconnected parameters, including the type of API, the type of formulation, and the penetration time. More research is needed to understand these effects in greater detail. For this, a more detailed characterization of the formulations—including high-resolution imaging, detailed analysis of the API location within the formulations, and higher numbers of biological replicates for each experiment (*n* = 6)—is suggested to further enhance the understanding of the effects and interactions observed.

The data from this study clearly demonstrate that PEVs and PlantCrystals were not beneficial for improving the dermal penetration efficacy of the hydrophilic API surrogate but proved to be beneficial for poorly water-soluble and lipophilic API surrogates. The likely reason is that hydrophilic APIs are not efficiently encapsulated into EVs but are rather distributed in the aqueous phase; consequently, they cannot be transported with the EVs. The lipophilic APIs—due to their lipophilicity—can be encapsulated into the EV structures (cf. Figure 1) and can thus be efficiently transported via the EVs. A high encapsulation rate can therefore be considered important for optimized dermal drug delivery.

The encapsulation of API also results in a slower penetration of the API surrogates, which was demonstrated for the PEVs and the loaded PlantCrystals, indicating that PEVs and PlantCrystals are also interesting drug-delivery systems for long-lasting therapeutic applications that require constant, controlled drug release. Possible indications would include anti-inflammatory APIs, antibiotics, and/or formulations for treating different kinds of pain. The use of PEVs and PlantCrystal would also be interesting for cosmetic and/or nutraceutical applications, for example, for improved delivery of antioxidants.

### 3.3. Influence of Milk Thistle PlantCrystals on Skin Hydration

**Figure 11:**
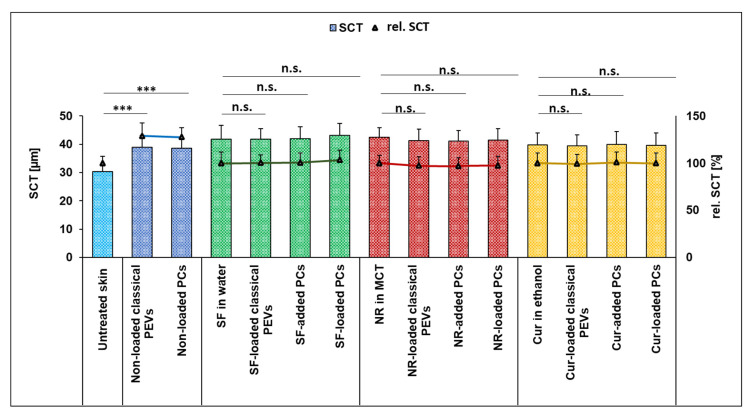
Stratum corneum thickness (SCT) (µm) and rel. SCT [%] of the different formulations (*** *p* < 0.001, n.s.: non-significant).

The final step of the study evaluated the effect of PlantCrystals on skin hydration. Adequate dermal hydration is essential for maintaining the skin barrier function, as dehydration can compromise barrier integrity and diminish the efficacy of topical treatments. Accordingly, the dermal formulation should promote sustained moisturization and prevent drying upon application. Milk thistle seeds contain various compounds that are considered beneficial for the skin (cf. Section 1). One major parameter that reflects the influence of a formulation on the skin properties is the measurement of the SCT, which indicates whether a formulation hydrates or dehydrates the skin [19]. In this study, the SCT was increased by about 30% (Dunn’s post hoc test, *p* < 0.001) after application of the PlantCrystals (Figure 11).

The addition of API led to a further increase in SCT (by about 10%, *p* < 0.05) for all formulations (Figure 11) and can be explained by the hygroscopic properties of the API, which attract water and thus further enhance skin hydration after they have penetrated into the stratum corneum and/or deeper layers. The results demonstrate the skin-hydrating properties of the silymarin PlantCrystals, making them not only highly effective but also a skin-friendly drug delivery system at the same time. Data could therefore confirm the hypothesis of the “2-in-1” benefit of the silymarin PlantCrystals.

## 4. Conclusions

Silymarin PlantCrystals were successfully produced and loaded with different types of API surrogates. The size of the unloaded PlantCrystals was about 300 nm, and loading with the APIs further increased the size of the formulations. The final size and size distribution of the PlantCrystals—due to the presence of fractured plant parts in the micrometer range—were rather large and broad. The type of API surrogate, the type of formulation, and the penetration time were found to be important parameters that influence the dermal penetration efficacy of the APIs. The penetration-enhancing effect—when compared to the other formulations—increased with increasing penetration times and was highest after 24 h of penetration, indicating a more controlled release of the APIs from the PlantCrystals when compared to classical API solutions. Effective dermal penetration enhancements were found for the lipophilic and poorly soluble API surrogates from the PlantCrystals when the APIs were added to the PlantCrystals prior to the milling process. The results confirmed findings from our previous studies and can be explained by the formation of EVs during the production of the PlantCrystals in which the APIs can be encapsulated. For the hydrophilic API surrogate, only a very minimal increase in penetration efficacy was found. This is because the hydrophilic API dissolves and distributes mainly in the aqueous dispersion medium, which hinders an efficient encapsulation into the more lipophilic EVs, thus hampering its efficient transportation into the skin with EVs. Silymarin PlantCrystals also improve skin hydration, thus making them a highly effective drug delivery system that is skin-friendly at the same time.

## Figures and Tables

**Table 1:** Overview of settings of microplate reader for the different types of API surrogates.

Type of API Surrogate	Excitation Wavelength (nm)	Emission Wavelength (nm)	Gain
Hydrophilic (SF)	550	580	94
Lipophilic (NR)	515	585	128
Poorly water soluble (Cur)	425	550	113

**Table 2:** Zeta potential [mV] of the different formulations. ZP values that were decreased when compared to the unloaded control are in bold letters.

	No API Surrogate in Formulation	Loaded with Hydrophilic API Surrogate (SF)	Loaded with Lipophilic API Surrogate (NR)	Loaded with Poorly Water-Soluble API Surrogate (Cur)
classical PEVs	−23 ± 1	−25 ± 3	−26 ± 4	**−21 ± 1**
PCs added	−25 ± 3	**−20 ± 2**	−28 ± 7	**−22 ± 4**
PCs loaded	−25 ± 3	−26 ± 3	−28 ± 3	**−19 ± 2**

## Data Availability

The original contributions presented in this study are included in the article/Supplementary Material. Further inquiries can be directed to the corresponding author.

## Supplementary Materials

The following supporting information can be downloaded at https://www.mdpi.com/article/10.3390/bioengineering12121331/s1, Table S1. Statistics for size analysis (DLS) data of different formulations. Table S2. Statistics for size analysis (LD) data of different formulations. Table S3. Statistics for penetration efficacy (MGV/Px) and rel. penetration efficacy [%] of different formulations. Table S4. Statistics for mean penetration depth [µm] and rel. MPD [%] of different formulations. Table S5. Statistics for time-dependent penetration efficacy (mg/ml) and rel. Penetration efficacy [%] of different formulations over 24 h. Table S6. Statistics for stratum corneum thickness (µm) and rel. SCT [%] of different formulations.

## Abbreviations

The following abbreviations are used in this manuscript:

API	Active pharmaceutical ingredient
EVs	Extracellular vesicles
Cur	Curcumin
MCT	Medium chain triglyceride (Miglyol^®^ 812)
MGV/px	Mean gray value/pixel
MPD	Mean penetration depth
NR	Nile red
PC	PlantCrystals
SCT	Stratum corneum thickness
SF	Sodium fluorescein
ZP	Zeta potential

## References

[1] Ebokaiwe A.P., Osawe S., Griffin S., Keck C.M., Olusanya O., Ehiri R.C. (2020). *Loranthus micranthus* nanoparticles abates streptozotocin-instigated testicular dysfunction in Wistar rats: Involvement of glucose metabolism enzymes, oxido-inflammatory stress, steroidogenic enzymes/protein and Nrf2 pathway. Andrologia.

[2] Griffin S., Sarfraz M., Farida V., Nasim M.J., Ebokaiwe A.P., Keck C.M., Jacob C. (2018). No time to waste organic waste: Nanosizing converts remains of food processing into refined materials. J. Environ. Manag..

[3] Sarfraz M., Griffin S., Gabour Sad T., Alhasan R., Nasim M.J., Irfan Masood M., Schäfer K.H., Ejike C.E.C.C., Keck C.M., Jacob C. (2018). Milling the Mistletoe: Nanotechnological Conversion of African Mistletoe (*Loranthus micranthus*) Intoantimicrobial Materials. Antioxidants.

[4] Griffin S., Alkhayer R., Mirzoyan S., Turabyan A., Zucca P., Sarfraz M., Nasim M.J., Trchounian A., Rescigno A., Keck C.M. (2017). Nanosizing *Cynomorium*: Thumbs up for Potential Antifungal Applications. Inventions.

[5] Abraham A.M., Wiemann S., Ambreen G., Zhou J., Engelhardt K., Brüßler J., Bakowsky U., Li S.-M., Mandic R., Pocsfalvi G. (2022). Cucumber-Derived Exosome-like Vesicles and PlantCrystals for Improved Dermal Drug Delivery. Pharmaceutics.

[6] Bhattacharya S., Preedy V.R., Watson R.R. (2020). Milk Thistle Seeds in Health. Nuts and Seeds in Health and Disease Prevention.

[7] Vostálová J., Tinková E., Biedermann D., Kosina P., Ulrichová J., Rajnochová Svobodová A. (2019). Skin Protective Activity of Silymarin and its Flavonolignans. Molecules.

[8] Singh R.P., Agarwal R. (2009). Cosmeceuticals and silibinin. Clin. Dermatol..

[9] Alkhaldi M., Sehra T., Sengupta S., Keck C.M. (2024). Extracellular Vesicles and Plant Crystals for Improved Bioavailability of Curcumin as a BCS Class IV Drug. Molecules.

[10] Romero G.B., Keck C.M., Müller R.H. (2016). Simple low-cost miniaturization approach for pharmaceutical nanocrystals production. Int. J. Pharm..

[11] Mota W.S., Severino P., Kadian V., Rao R., Zielińska A., Silva A.M., Mahant S., Souto E.B. (2025). Nanometrology: Particle sizing and influence on the toxicological profile. Front. Nanotechnol..

[12] Hoshyar N., Gray S., Han H., Bao G. (2016). The effect of nanoparticle size on in vivo pharmacokinetics and cellular interaction. Nanomedicine.

[13] Joudeh N., Linke D. (2022). Nanoparticle classification, physicochemical properties, characterization, and applications: A comprehensive review for biologists. J. Nanobiotechnol..

[14] Pelikh O., Pinnapireddy S.R., Keck C.M. (2021). Dermal Penetration Analysis of Curcumin in an ex vivo Porcine Ear Model Using Epifluorescence Microscopy and Digital Image Processing. Skin Pharmacol. Physiol..

[15] Keck C.M., Abdelkader A., Pelikh O., Wiemann S., Kaushik V., Specht D., Eckert R.W., Alnemari R.M., Dietrich H., Brüßler J. (2022). Assessing the Dermal Penetration Efficacy of Chemical Compounds with the Ex-Vivo Porcine Ear Model. Pharmaceutics.

[16] Abraham A.M., Alnemari R.M., Jacob C., Keck C.M. (2020). PlantCrystals-Nanosized Plant Material for Improved Bioefficacy of Medical Plants. Materials.

[17] Abraham A.M., Alnemari R.M., Brüßler J., Keck C.M. (2021). Improved Antioxidant Capacity of Black Tea Waste Utilizing PlantCrystals. Molecules.

[18] Abraham A.M., Quintero C., Carrillo-Hormaza L., Osorio E., Keck C.M. (2021). Production and Characterization of Sumac PlantCrystals: Influence of High-Pressure Homogenization on Antioxidant Activity of Sumac (*Rhus coriaria* L.). Plants.

[19] Kaushik V., Ganashalingam Y., Schesny R., Raab C., Sengupta S., Keck C.M. (2021). Influence of Massage and Skin Hydration on Dermal Penetration Efficacy of Nile Red from Petroleum Jelly-An Unexpected Outcome. Pharmaceutics.

