# Supplementary material for: Silymarin PlantCrystals for Improved Dermal Drug Delivery
Source: Bioengineering (Basel). 2025 Dec 5;12(12):1331. doi: 10.3390/bioengineering12121331 (PMC12729838; doi:10.3390/bioengineering12121331)
Supplement: Supplementary file 1 [file bioengineering-12-01331-s001.zip › bioengineering-3952464-supplementary.pdf]

SUPPLEMENTARY MATERIAL

Table S1. Statistics for size analysis (DLS) data of different formulations
(Dunn's post-hoc comparison of z-average [nm])
(** p < .01, *** p < .001, n.s.: non-significant)

<table>
<tr><th></th><th>Non-loaded classical PEVs</th><th>Non-loaded PCs</th><th>Among added and loaded PCs</th></tr>
<tr><td>SF-loaded classical PEVs</td><td>***</td><td>-</td><td>-</td></tr>
<tr><td>SF-added PCs</td><td>-</td><td>**</td><td rowspan="2">**</td></tr>
<tr><td>SF-loaded PCs</td><td></td><td>n.s.</td></tr>
<tr><td>NR-loaded classical PEVs</td><td>**</td><td>-</td><td></td></tr>
<tr><td>NR-added PCs</td><td>-</td><td>***</td><td rowspan="2">n.s.</td></tr>
<tr><td>NR-loaded PCs</td><td>-</td><td>**</td></tr>
<tr><td>Cur-loaded classical PEVs</td><td>***</td><td>-</td><td>-</td></tr>
<tr><td>Cur-added PCs</td><td>-</td><td>***</td><td rowspan="2">**</td></tr>
<tr><td>Cur-loaded PCs</td><td>-</td><td>**</td></tr>
</table>

Table S2. Statistics for size analysis (LD) data of different formulations
(Dunn's post-hoc comparison of d(v) 0.95 of all formulations)
(** p < .01, *** p < .001, n.s.: non-significant)

<table>
<tr><th></th><th>Non-loaded PCs</th><th>Among added and loaded PCs</th></tr>
<tr><td>SF-added PCs</td><td>***</td><td rowspan="2">**</td></tr>
<tr><td>SF-loaded PCs</td><td>***</td></tr>
<tr><td>NR-added PCs</td><td>***</td><td rowspan="2">n.s.</td></tr>
<tr><td>NR-loaded PCs</td><td>***</td></tr>
<tr><td>Cur-added PCs</td><td>n.s.</td><td rowspan="2">**</td></tr>
<tr><td>Cur-loaded PCs</td><td>***</td></tr>
</table>

Table S3. Statistics for penetration efficacy (MGV/Px) and rel. penetration efficacy [%] of different formulations
(Dunn's post-hoc comparison of Penetration efficacy (MGV/Px) of the formulations)
(* p < .05, *** p < .001, n.s.: non-significant)

	SF in water	NR in MCT	Cur in ethanol
SF-loaded classical PEVs	***	-	-
SF-added PCs	n.s.	-	-
SF-loaded PCs	n.s.	-	-
NR-loaded classical PEVs	-	***	-
NR-added PCs	-	*	-
NR-loaded PCs	-	***	-
Cur-loaded classical PEVs	-	-	***
Cur-added PCs	-	-	***
Cur-loaded PCs	-	-	***

Table S4. Statistics for mean penetration depth [µm] and rel. MPD [%] of different formulations
(Dunn's post-hoc comparison of mean penetration depth [µm] of the formulations)
(* p < .05, ** p < .01, *** p < .001, n.s.: non-significant)

	SF in water	NR in MCT	Cur in ethanol
SF-loaded classical PEVs	***	-	-
SF-added PCs	n.s.	-	-
SF-loaded PCs	n.s.	-	-
NR-loaded classical PEVs	-	**	-
NR-added PCs	-	***	-
NR-loaded PCs	-	***	-
Cur-loaded classical PEVs	-	-	***
Cur-added PCs	-	-	***
Cur-loaded PCs	-	-	*

Table S5. Statistics for time-dependent penetration efficacy (mg/ml) and rel. Penetration efficacy [%] of different formulations over 24 h.
(Dunn's post-hoc comparison of benchmark control and classical PEVs and PC formulations)
(* p < .05, ** p < .01, n.s.: non-significant)

	SF in water					NR in MCT					Cur in ethanol				
	15 min	2h	6h	12h	24h	15 min	2h	6h	12h	24h	15 min	2h	6h	12h	24h
SF-loaded classical PEVs	*	n.s.	n.s.	n.s.	n.s.	-	-	-	-	-	-	-	-	-	-
SF-added PCs	n.s.	n.s.	n.s.	n.s.	n.s.	-	-	-	-	-	-	-	-	-	-
SF-loaded PCs	n.s.	n.s.	n.s.	n.s.	n.s.	-	-	-	-	-	-	-	-	-	-
NR-loaded classical PEVs	-	-	-	-	-	**	*	*	n.s.	n.s.	-	-	-	-	-
NR-added PCs	-	-	-	-	-	n.s.	n.s.	n.s.	n.s.	n.s.	-	-	-	-	-
NR-loaded PCs	-	-	-	-	-	n.s.	n.s.	n.s.	*	n.s.	-	-	-	-	-
Cur-loaded classical PEVs	-	-	-	-	-	-	-	-	-	**	n.s.	n.s.	n.s.	n.s.	n.s.
Cur-added PCs	-	-	-	-	-	-	-	-	-	-	n.s.	n.s.	n.s.	n.s.	n.s.
Cur-loaded PCs	-	-	-	-	-	-	-	-	-	-	n.s.	n.s.	n.s.	*	*

Table S6. Statistics for stratum corneum thickness (µm) and rel. SCT [%] of different formulations

(Dunn's post-hoc comparison of stratum corneum thickness (µm) of the formulations)

(* p < .05, ** p < .01, *** p < .001, n.s.: non-significant)

	Untreated skin	SF in water	NR in MCT	Cur in ethanol
Non-loaded classical PEVs	***	-	-	-
Non-loaded PCs	***	-	-	-
SF-loaded classical PEVs	-	n.s.	-	-
SF-added PCs	-	n.s.	-	-
SF-loaded PCs	-	n.s.	-	-
NR-loaded classical PEVs	-	-	n.s.	-
NR-added PCs	-	-	n.s.	-
NR-loaded PCs	-	-	n.s.	-
Cur-loaded classical PEVs	-	-	-	n.s.
Cur-added PCs	-	-	-	n.s.
Cur-loaded PCs	-	-	-	n.s.
